# Supplementary figures and images for: A protease-activatable luminescent biosensor and reporter cell line for authentic SARS-CoV-2 infection
Source: PLoS Pathog. 2022 Feb 10;18(2):e1010265. doi: 10.1371/journal.ppat.1010265 (PMC8865646; doi:10.1371/journal.ppat.1010265)

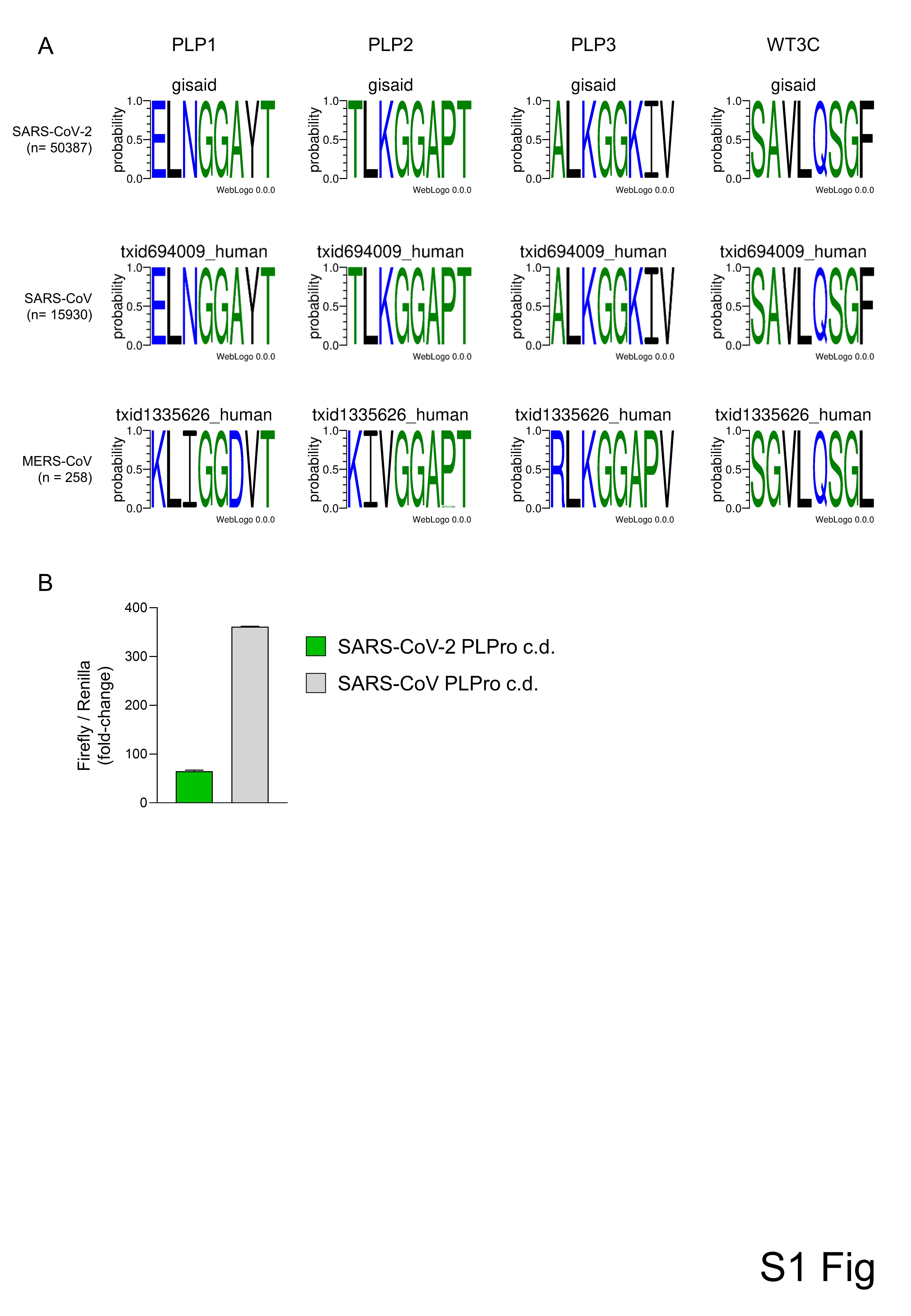

Supplement: S1 Fig — (A) Conservation of cleavage sequences. Amino acid sequence logos for cleavage sequences between nsp1/nsp2 (PLP1), nsp2/nsp3 (PLP2), nsp3/nsp4 (PLP3), and nsp4/nsp5 (WT3c) of SARS-CoV-2, SARS-CoV and MERS-CoV viruses. Relative letter heights indicate conservation across the sequences analysed. (B) Luminescent biosensor activation by PLPro of SARS-CoV. HEK293T cells were co-transfected with the 30F-PLP2 biosensor and the catalytic domain of recombinant Papain-Like Protease (PLPro c.d.) of either SARS-CoV-2 or SARS-CoV. Firefly and Renilla luciferase activities were measured by luminometry 24 h post-transfection. Mean fold-changes in Firefly/Renilla luminescence ± SEM in the presence or absence of protease are shown for an experiment performed in triplicate. Representative of 2 independent experiments. (JPG) [file ppat.1010265.s001.jpg]

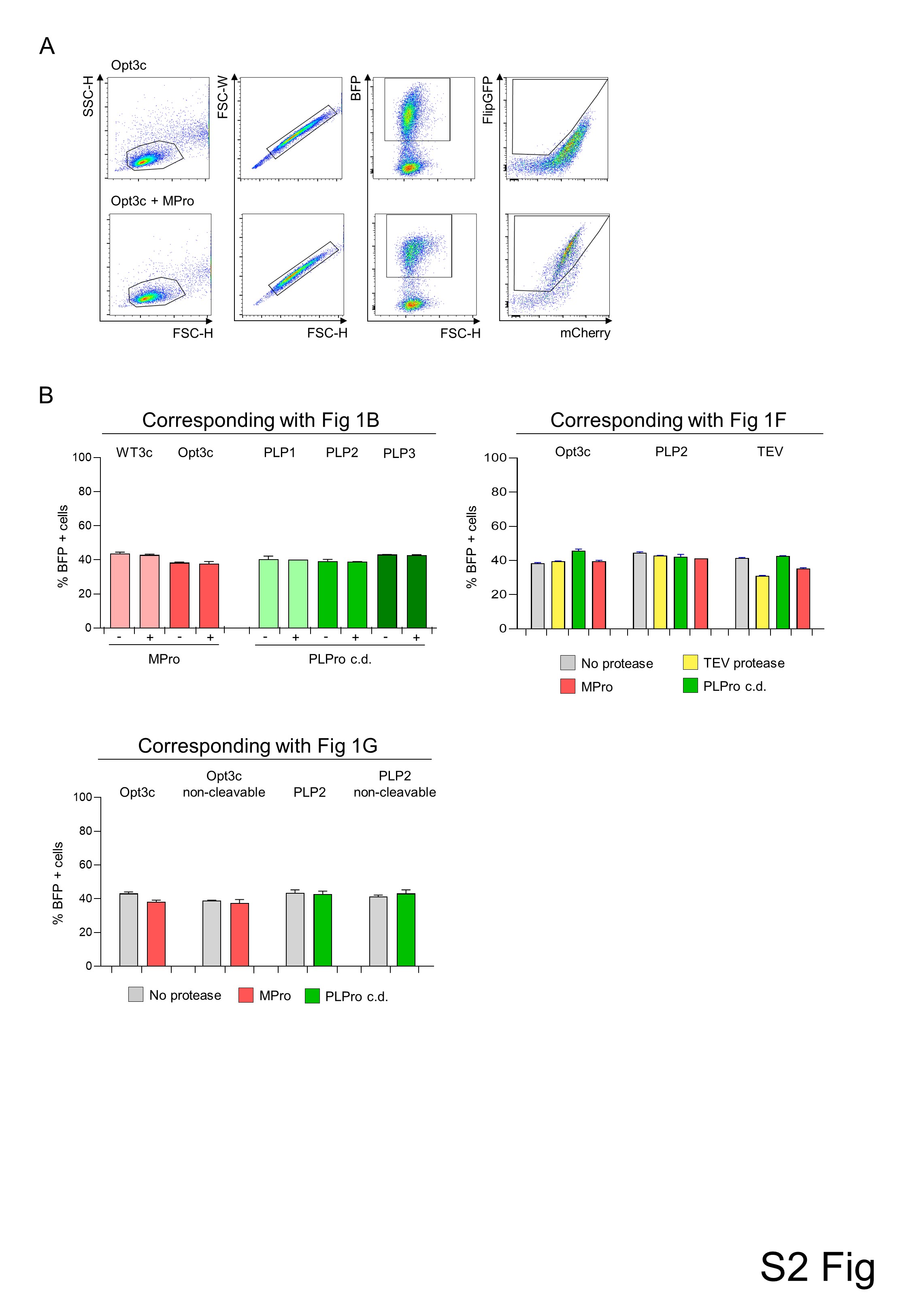

Supplement: S2 Fig — (A) Indicative gating strategy. Cells were typically gated on FSC-H and SSC-H, and doublets excluded using FSC-W. FlipGFP and mCherry fluorescence were analysed in BFP+ (transfected) cells. Data illustrate change in fluorescence of Opt3c-FlipGFP biosensor in the presence (lower panels) or absence (upper panels) of Main Protease (MPro). (B) Transfection efficiency. % BFP+ (transfected) cells for experiments in Fig 1B, 1F and 1G are displayed. Mean values ± SEM are shown for experiments performed in triplicate, representative of at least 3 independent experiments. (JPG) [file ppat.1010265.s002.jpg]

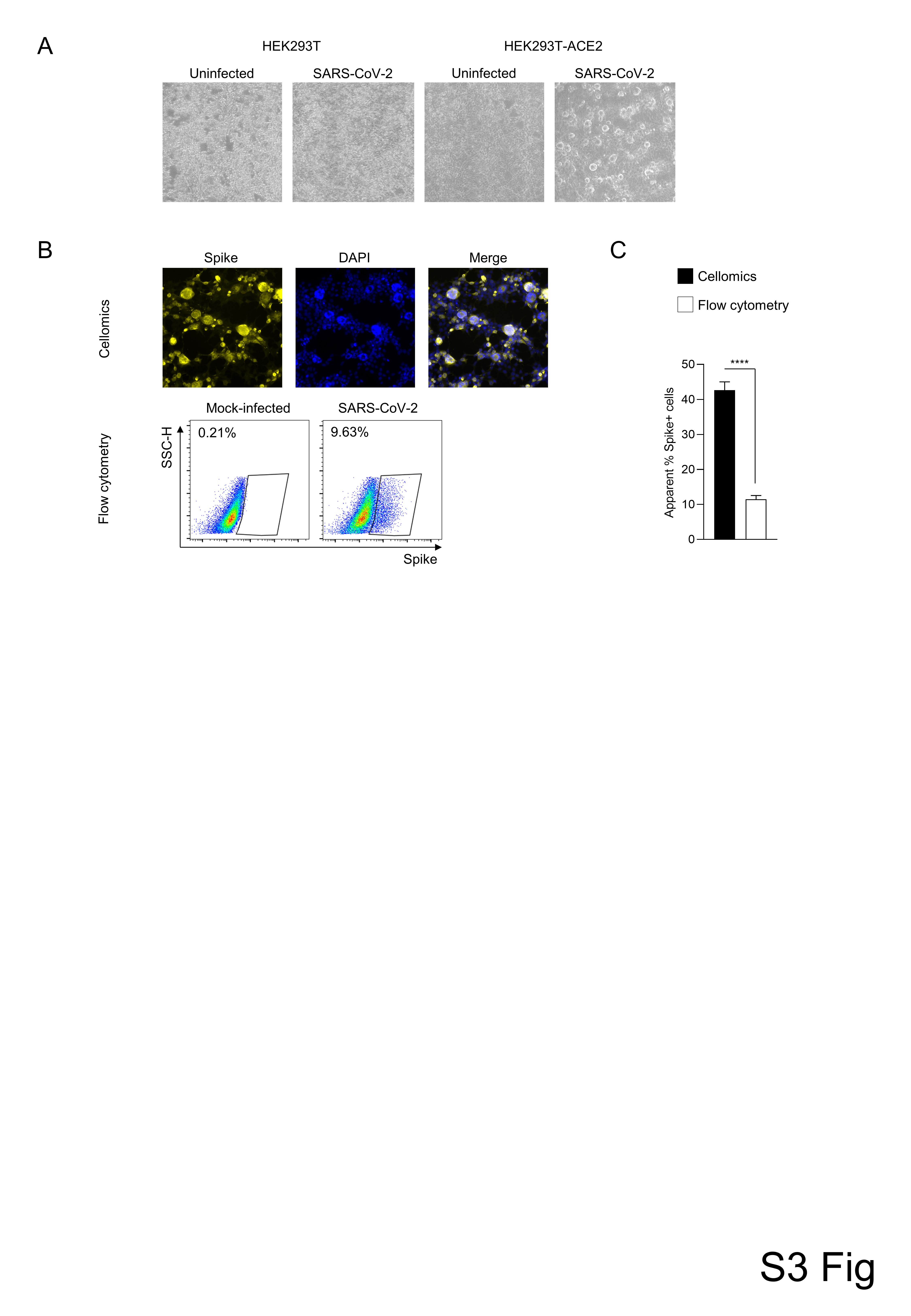

Supplement: S3 Fig — (A) Cytopathic effect in HEK293T-ACE2 cells. HEK293T or HEK293T-ACE2 cells were infected with SARS-CoV-2 at MOI = 1 then examined for cytopathic effect using brightfield microscopy after 24 h. Representative of >10 independent experiments. (B-C) Loss of spike+ HEK293T-ACE2 cells during flow cytometric analysis. HEK293T-ACE2 cells were infected with SARS-CoV-2 at MOI = 1. Cells were analysed in parallel 24 h post-infection for SARS-CoV-2 spike protein by either automated microscopy (Cellomics) or flow cytometry. In each case, the proportion of spike+ cells was measured. Illustrative data (B) and mean values ± SEM (C) are shown for an experiment performed in triplicate. **** p<0.0001. Spike, yellow. DAPI, blue. Representative of 2 independent experiments. (JPG) [file ppat.1010265.s003.jpg]

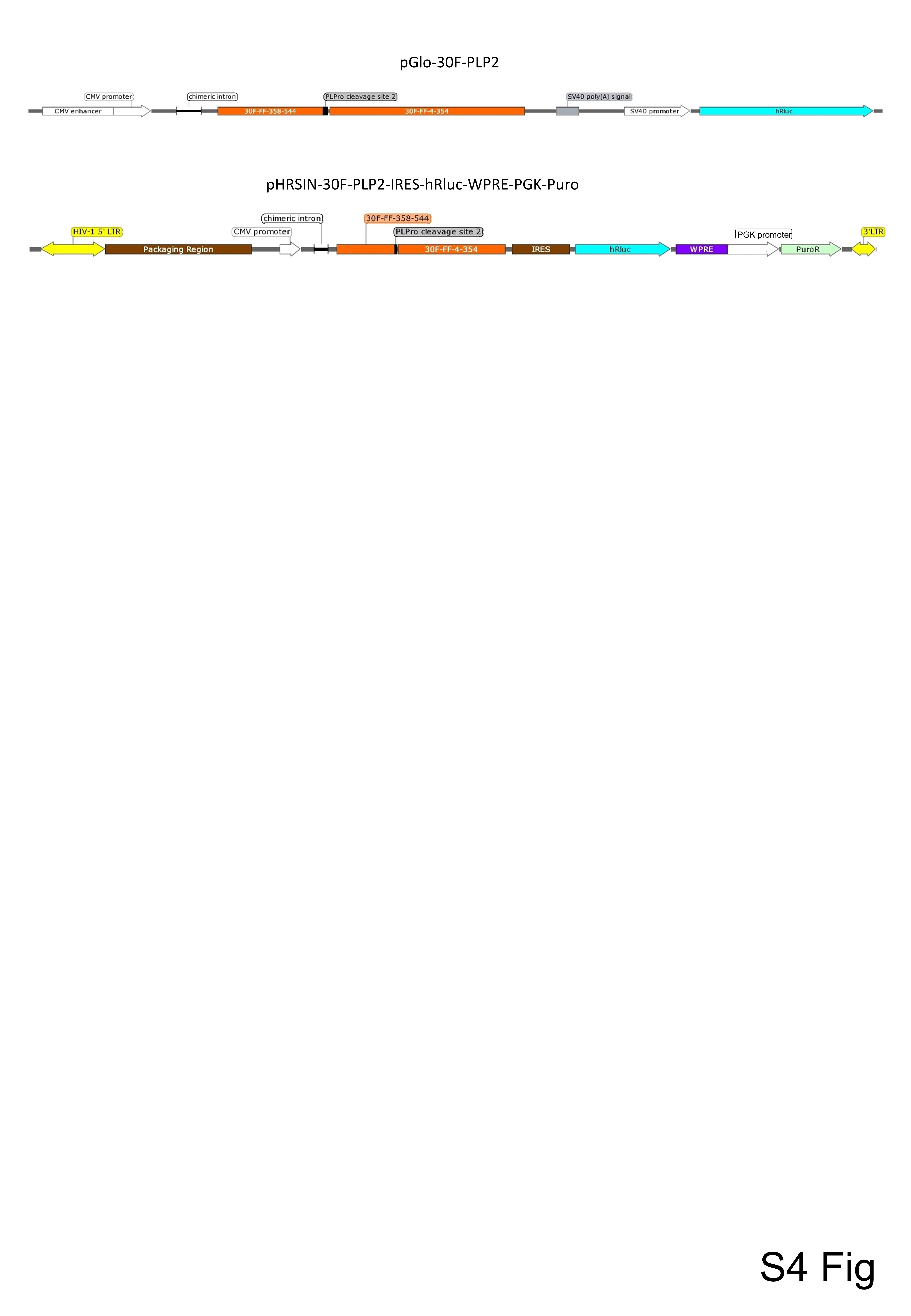

Supplement: S4 Fig — Diagrams of 30F-PLP2 luciferase-based reporter in pGloSensor-30F and pHRSIN-30F-PLP2-IRES-hRluc-WPRE-PGK-Puro expression vectors. 30-FF-358-544/30-FF-4-354, circularly permuted firefly luciferase-based reporter, orange (numbers indicate positions of amino acids in wildtype FFluc). hRluc, codon-optimised (humanised) Renilla luciferase, cyan. LTR, HIV-1 long terminal repeat, yellow. IRES, internal ribosome entry site, brown. WPRE, woodchuck hepatitis virus post-transcriptional regulatory element (WPRE), purple. PuroR, puromycin resistance, green. (JPG) [file ppat.1010265.s004.jpg]

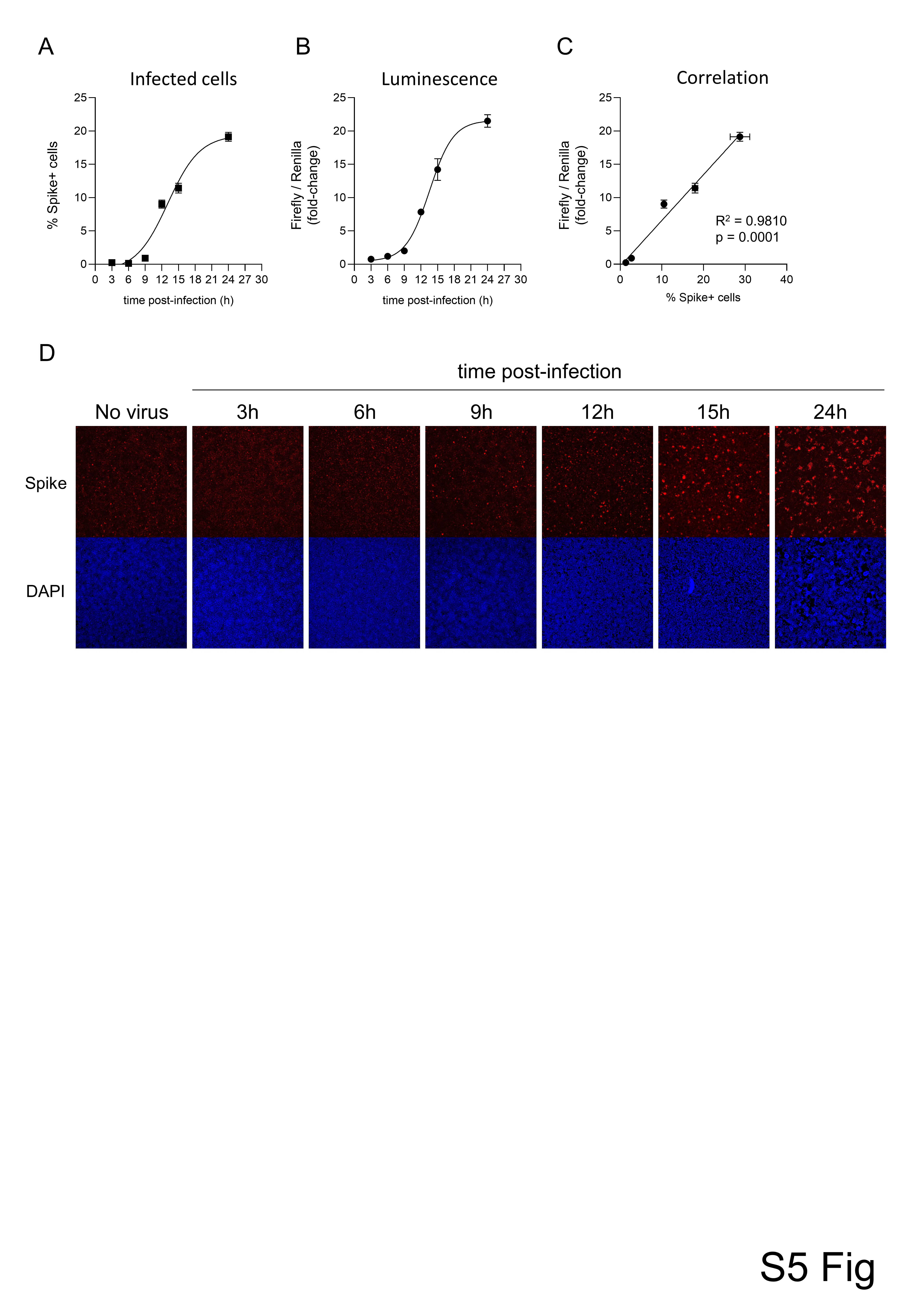

Supplement: S5 Fig — (A-D) Clone B7 reporter cells were mock-infected or infected with SARS-CoV-2 at MOI = 0.01 and analysed in parallel at the indicated time points post-infection by either epifluorescence microscopy for SARS-CoV-2 spike protein (A), or luminometry for Firefly and Renilla Luciferase activities (B). Spike+ cells were enumerated by automated microscopy (Cellomics). Firefly/Renilla luminescence is shown as fold-change with/without infection. Mean values ± SEM (A-C) are shown for an experiment performed in triplicate, together with illustrative microscopy data (D). The correlation between the ratio of Firefly/Renilla luminescence and the proportions of spike+ cells is shown in C. Spike, red. DAPI, blue. R2, Pearson’s correlation coefficient. Representative of 2 independent experiments. (JPG) [file ppat.1010265.s005.jpg]

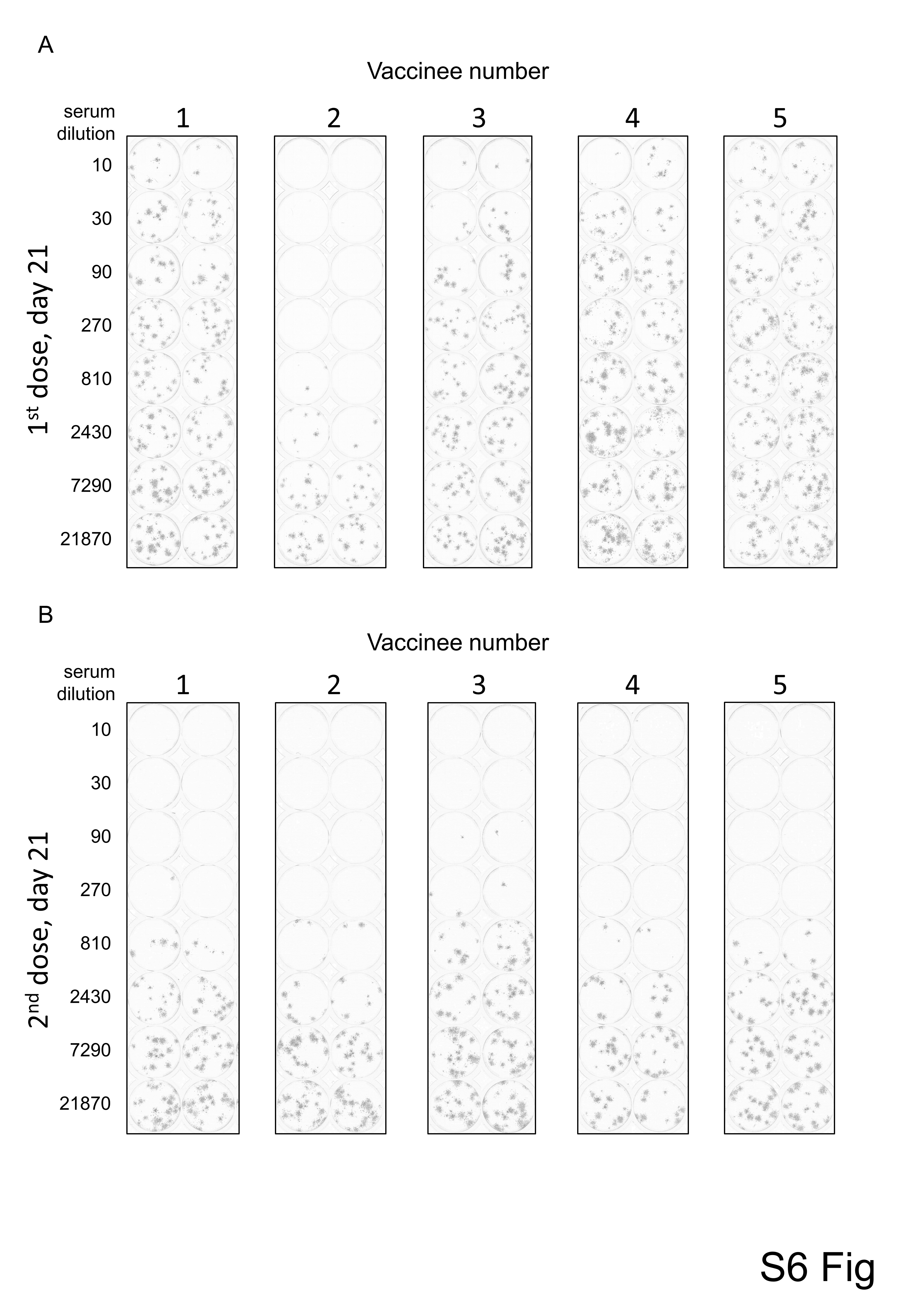

Supplement: S6 Fig — (A-B) Illustrative PRNT data from Fig 5A. SARS-CoV-2-ZsGreen viral stock (MOI = 0.001) was pre-incubated with a 3-fold dilution series of heat-inactivated serum from 5 healthy control donors 21 days after their 1st dose (A) or 2nd dose (B) of Pfizer-BioNTech BNT162b2 mRNA vaccine before addition to VeroE6 cells. A semi-solid overlay was added after 2 h, and cells fixed and analysed by automated microscopy (Cellomics) 48 h post-infection. For each well, 36 independent images were acquired and stitched together using Fiji (ImageJ). ZsGreen fluorescence is shown in grey. (JPG) [file ppat.1010265.s006.jpg]

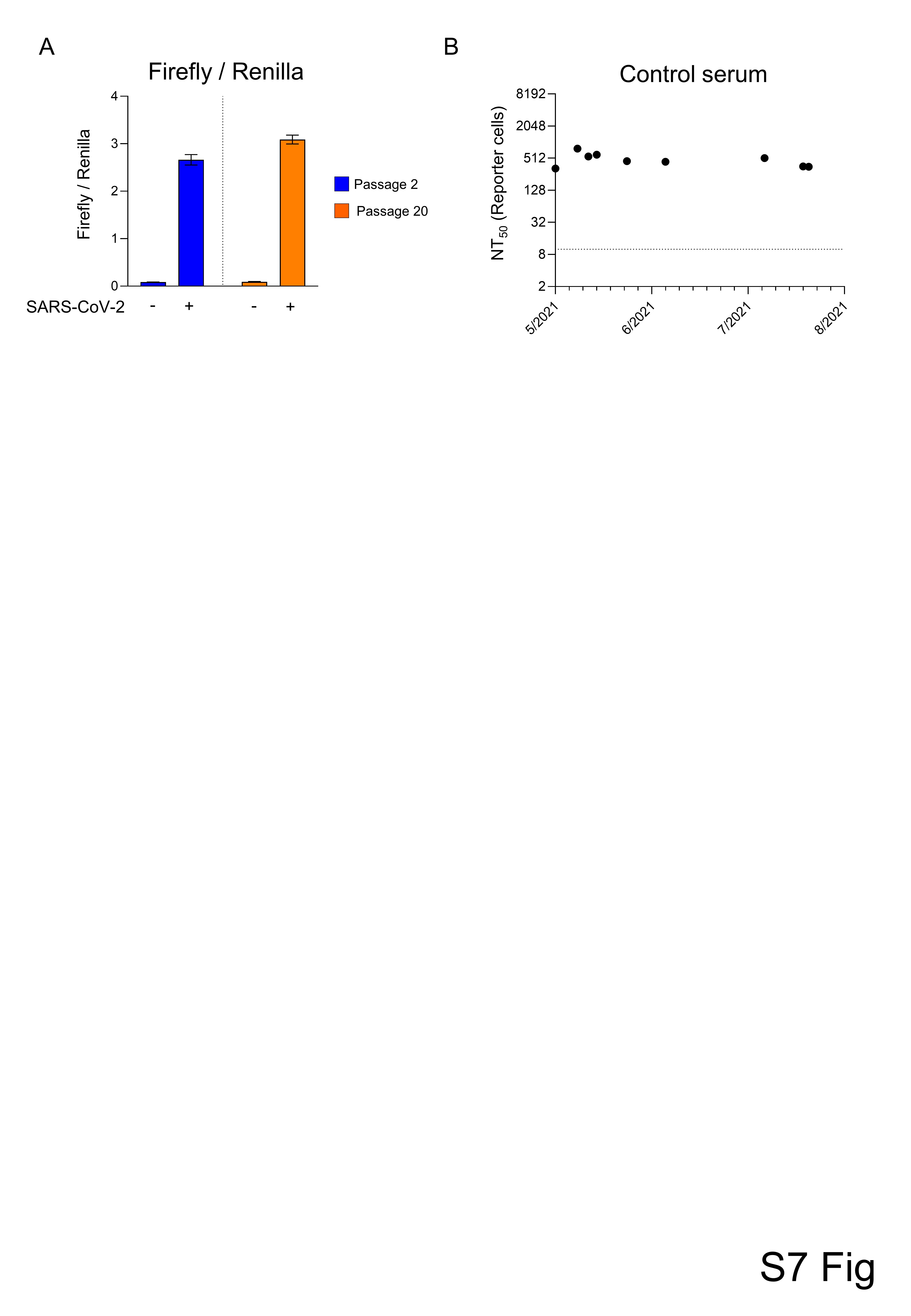

Supplement: S7 Fig — (A) Comparison of luminescent biosensor activation in low passage vs. high passage reporter cells. The first freeze of clone B7 reporter cells (Passage 2) and cells kept in culture for two months (Passage 20) were infected with SARS-CoV-2 at MOI = 0.01. Firefly and Renilla luciferase activities were measured by luminometry 24 h post-infection. Mean Firefly/Renilla luminescence ± SEM are shown for an experiment performed in triplicate. Representative of 2 independent experiments. (B) Reproducibility of NT50s obtained using clone B7 reporter cells. The serum from a healthy control donor obtained 21 days after their 2nd dose of Pfizer-BioNTech BNT162b2 mRNA vaccine was frozen in single-use (20 μL) aliquots at -80°C and routinely included as an internal control in all assays of SARS-CoV-2 neutralising activity. NT50s were determined using clone B7 reporter cells as shown in Fig 5A. Data are shown for 9 independent experiments conducted in duplicate over a period of three months. The dotted line indicates the limit of detection (lowest dilution) for the assay. (JPG) [file ppat.1010265.s007.jpg]

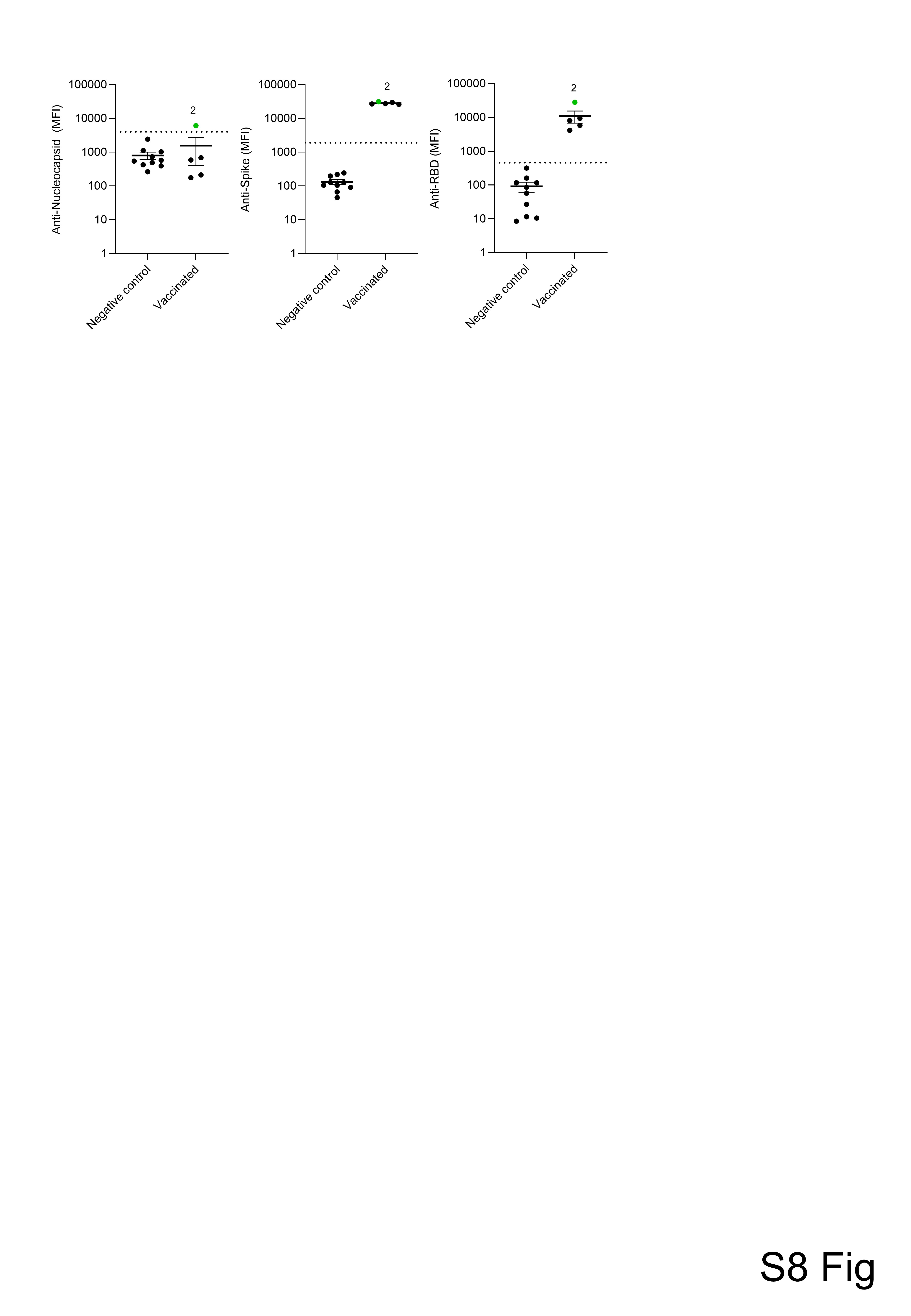

Supplement: S8 Fig — Antibodies to SARS-CoV-2 nucleocapsid, trimeric spike and spike receptor-binding domain were quantitated by Luminex bead-based immunoassay. Negative controls, stored serum samples collected prior to 2019. Vaccinated, serum samples from 5 healthy control donors 21 days after their 1st dose of Pfizer-BioNTech BNT162b2 mRNA vaccine (same serum samples as Fig 5A, upper panels). Donor 2 is highlighted in green. Dotted lines indicate diagnostic cut-offs determined by receiver operating characteristic (ROC) curve analysis. MFI, mean fluorescence intensity. (JPG) [file ppat.1010265.s008.jpg]
