# Supplementary material for: A protease-activatable luminescent biosensor and reporter cell line for authentic SARS-CoV-2 infection
Source: PLoS Pathog. 2022 Feb 10;18(2):e1010265. doi: 10.1371/journal.ppat.1010265 (PMC8865646; doi:10.1371/journal.ppat.1010265)
Supplement: S1 Table — (PDF) [file ppat.1010265.s009.pdf]

S1 Table. PCR primers for generation of FlipGFP-based reporters.

Construct	Primer	Sequence[1,2]
WT3c (PCR1)	Near AfeI	GCGGTCTCGAGATGGAAGTTAGC
	WT3c Rv	AAACCCGGATTGCAGTACGGCACTTGATGCATCGGTAATGCCAG
WT3c (PCR2)	WT3c Fw	AGTGCCGTACTGCAATCCGGGTTTAAGGTGTCCGCCCTGAAGGA
	Near AflII	CTCTGCCCTCGATATCCTCC
Opt3c (PCR1)	Near AfeI	GCGGTCTCGAGATGGAAGTTAGC
	Opt3c Rv	GAAACCAGACTGGAGCCGAACCGTTGATGCATCGGTAATGCCAG
Opt3c (PCR2)	Opt3c Fw	ACGGTTCGGCTCCAGTCTGGTTTCAAGGTGTCCGCCCTGAAGGA
	Near AflII	CTCTGCCCTCGATATCCTCC
PLP1 (PCR1)	Near AfeI	GCGGTCTCGAGATGGAAGTTAGC
	PLP1 Rv	GGTGTAGGCACCGCCATTAAGTTCTGATGCATCGGTAATGCCAG
PLP1 (PCR2)	PLP1 Fw	GAACTTAATGGCGGTGCCTACACCAAGGTGTCCGCCCTGAAGGA
	Near AflII	CTCTGCCCTCGATATCCTCC
PLP2 (PCR1)	Near AfeI	GCGGTCTCGAGATGGAAGTTAGC
	PLP2 Rv	AGTAGGTGCTCCTCCCTTCAGTGTTGATGCATCGGTAATGCCAG
PLP2 (PCR2)	PLP2 Fw	ACACTGAAGGGAGGAGCACCTACTAAGGTGTCCGCCCTGAAGGA
	Near AflII	CTCTGCCCTCGATATCCTCC
PLP3 (PCR1)	Near AfeI	GCGGTCTCGAGATGGAAGTTAGC
	PLP3 Rv	GACGATCTTTCCACCTTTCAAGGCTGATGCATCGGTAATGCCAG
PLP3 (PCR2)	PLP3 Fw	GCCTTGAAAGGTGGAAAGATCGTCAAGGTGTCCGCCCTGAAGGA
	Near AflII	CTCTGCCCTCGATATCCTCC
Non-cleavable Opt3c (PCR1)	Near AfeI	GCGGTCTCGAGATGGAAGTTAGC
	Opt3c CR Rv	GAAACCAGA**TAT**GAGCCGAACCGTTGATGCATCGGTAATGCCAG
Non-cleavable Opt3c (PCR2)	Opt3c CR Fw	ACGGTTCGGCTC**ATA**TCTGGTTTCAAGGTGTCCGCCCTGAAGGA
	Near AflII	CTCTGCCCTCGATATCCTCC
Non-cleavable PLP2 (PCR1)	Near AfeI	CTCTGCCCTCGATATCCTCC
	PLP2 CR Rv	AGTAGGTGC**CTTTCCCAGTCC**TGTTGATGCATCGGTAATGCCAG
Non-cleavable PLP2 (PCR2)	PLP2 CR Fw	ACA**GGACTGGGAAAG**GCACCTACTAAGGTGTCCGCCCTGAAGGA
	Near AflII	GCGGTCTCGAGATGGAAGTTAGC

1. Codons encoding oligopeptide cleavage sequences shaded in grey.
2. Mutated sequences in cleavage-resistant reporters highlighted in bold.
