# Supplementary material for: A protease-activatable luminescent biosensor and reporter cell line for authentic SARS-CoV-2 infection
Source: PLoS Pathog. 2022 Feb 10;18(2):e1010265. doi: 10.1371/journal.ppat.1010265 (PMC8865646; doi:10.1371/journal.ppat.1010265)
Supplement: S2 Table — (PDF) [file ppat.1010265.s010.pdf]

S2 Table. Oligonucleotides for generation of luciferase-based reporters.

Construct	Oligo	Sequence[1,2]
30F-Opt3c	Fw	CAAATGTTAGAAAAACACACCAATGGATCAACGGTTCGGCTCCAGTCTGGTTTCGGAAGTTTGAAAAACATCCTGTATGGTC
	Rv	GACCATACAGGATGTTTTTCAAACTTCCGAAACCAGACTGGAGCCGAACCGTTGATCCATTGGTGTGTTTTTCTAACATTTG
Non-cleavable 30F-Opt3c	Fw	CAAATGTTAGAAAAACACACCAATGGATCAACGGTTCGGCTC**ATA**TCTGGTTTCGGAAGTTTGAAAAACATCCTGTATGGTC
	Rv	GACCATACAGGATGTTTTTCAAACTTCCGAAACCAGA**TAT**GAGCCGAACCGTTGATCCATTGGTGTGTTTTTCTAACATTTG
30F-PLP2	Fw	CAAATGTTAGAAAAACACACCAATGGATCAACACTGAAGGGAGGAGCACCTACTGGAAGTTTGAAAAACATCCTGTATGGTC
	Rv	GACCATACAGGATGTTTTTCAAACTTCCAGTAGGTGCTCCTCCCTTCAGTGTTGATCCATTGGTGTGTTTTTCTAACATTTG
Non-cleavable 30F-PLP2	Fw	CAAATGTTAGAAAAACACACCAATGGATCAACA**GGACTGGGAAAG**GCACCTACTGGAAGTTTGAAAAACATCCTGTATGGTC
	Rv	GACCATACAGGATGTTTTTCAAACTTCCAGTAGGTGC**CTTTCCCAGTCC**TGTTGATCCATTGGTGTGTTTTTCTAACATTTG

1. Codons encoding oligopeptide cleavage sequences shaded in grey.
2. Mutated sequences in cleavage-resistant reporters highlighted in bold.
