# Supplementary material for: A protease-activatable luminescent biosensor and reporter cell line for authentic SARS-CoV-2 infection
Source: PLoS Pathog. 2022 Feb 10;18(2):e1010265. doi: 10.1371/journal.ppat.1010265 (PMC8865646; doi:10.1371/journal.ppat.1010265)
Supplement: S1 Appendix — (PDF) [file ppat.1010265.s011.pdf]

S1 Appendix. Complete sequence of 30F-PLP2-IRES-hRluc-WPRE-PGK-Puro reporter cassette.

GGCATTGATTATTGACTAGTTATTAATAGTAATCAATTACGGGGTCATTAGTTCATAGCCCATATAT
GGAGTTCCGCGTTACATAACTTACGGTAAATGGCCCGCCTGGCTGACCGCCCAACGACCCCCG
CCCATTGACGTCAATAATGACGTATGTTCCCATAGTAACGCCAATAGGGACTTTCCATTGACGTC
AATGGGTGGAGTATTTACGGTAAACTGCCCACTTGGCAGTACATCAAGTGTATCATATGCCAAGT
CCGCCCCCTATTGACGTCAATGACGGTAAATGGCCCGCCTGGCATTATGCCCAGTACATGACCT
TACGGGACTTTCCTACTTGGCAGTACATCTACGTATTAGTCATCGCTATTACCATGGTGATGCGG
TTTTGGCAGTACACCAATGGGCGTGGATAGCGGTTTGACTCACGGGGATTTCCAAGTCTCCACC
CCATTGACGTCAATGGGAGTTTGTTTTGGCACCAAAATCAACGGGACTTTCCAAAATGTCGTAAT
AACCCCGCCCCGTTGACGCAAATGGGCGGTAGGCGTGTACGGTGGGAGGTCTATATAAGCAGA
GC[1]TGGTTTAGTGAACCGTCAGATCACTAGAAGGTTTATTGCGGTAGTTTATCACAGTTAAATTGC
TAACGCAGTCAGTGCTTCTGACACAACAGTCTCGAACTTAAGCTGCAGAAGTTGGTCGTGAGGC
ACTGGGCAGGTAAGTATCAAGGTTACAAGACAGGTTTAAGGAGACCAATAGAAACTGGGCTTGT
CGAGACAGAGAAGACTCTTGCGTTTCTGATAGGCACCTATTGGTCTTACTGACATCCACTTTGCC
TTTCTCTCCACAG[2]GTGTCCACTCCCAGTTCAATTACAGCTCTTAAGGCTAGAGTATTAATACGAC
TCACTATAGGGAATTTTAACTTTACTAAGGAGCGATCGCCATGCCGGGATCAACTGGTAAAATAG
TACCATTACACGCTGTTAAAGTTGTCGATCCTACAACAGGAAAAATTTTGGGGCCAAATGAACCT
GGAGAATTGTATTTTAAAGGCCCGATGATAATGAAGGGTTATTATAATAATGAAGAAGCTACTAAA
GCAATTATTGATAATGACGGATGGTTGCGCTCTGGTGATATTGCTTATTATGACAATGATGGCCAT
TTTTATATTGTGGACAGGCTGAAGTCACTGATTAAATATAAAGGTTATCAGGTTGCACCTGCTGAA
ATTGAGGGAATACTCTTACAACATCCGTATATTGTTGATGCCGGCGTTACTGGTATACCGGATGA
AGCCGCGGGCGAGCTTCCAGCTGCAGGTGTTGTAGTACAGACTGGAAAATATCTAAACGAACAA
ATCGTACAAGATTATGTTGCCAGTCAAGTTTCAATAGCCAAATGGCTACGTGGTGGGGTGAAATT
TTTGGATGAAATTCCCAAAGGATCAACTGGAAAAATTGACAGAAAAGTGTTAAGACAAATGTTAGA
AAAACACACCAATGGA[3]TCAACACTGAAGGGAGGAGCACCTACT[4]GGAAGTTTGAAAAACATCCT
GTATGGTCCGGAACCGTTCTACCCACTGGAAGATGGTACCGCTGGTGAACAGATGTTTGACGCA
TTATCTCGTTATGCAGCTATTCCGGGCTGCATAGCATTGACAAATGCTCATACAAAAGAAAATGTT
TTATATGAAGAGTTTCTGAAACTGTCGTGTCGTTTAGCGGAAAGTTTTAAAAAGTATGGATTAAAA
CAAAACGACACAATAGCGGTGTGTAGCGAAAATAGTCTGCAATTTTTCCTTCCTGTAATTGCATCA
TTGTATCTTGGAATAATTGTGGCACCTGTTAACGATAAATACATTGAACGTGAATTAATACACAGT
CTTGGTATTGTAAAACCACGCATAGTTTTTTGCTCCAAGAATACTTTTCAAAAAGTACTGAATGTAA
AATCTAAATTAAAATCTATTGAAACTATTATTATATTAGACTTAAATGAAGACTTAGGAGGTTATCA
ATGCCTCAACAACTTTATTTCTCAAAATTCCGATAGTAATCTGGACGTAAAAAAATTTAAACCCTAT
TCTTTTAATCGAGACGATCAGGTTGCGTCGATTATGTTTTCTTCTGGTACAACTGGTCTGCCGAA
GGGAGTCATGCTAACTCACAAGAATATTGTTGCACGATTTTCTATTGCAAAAGATCCTACTTTTGG
TAACGCAATTAATCCCACGTCAGCAATTTTAACGGTAATACCTTTCCACCATGGTTTTGGTATGAT
GACCACATTAGGATACTTTACTTGTGGATTCCGAGTTGTTCTAATGCACACGTTTGAAGAAAACT
ATTTCTACAATCATTACAAGATTATAAAGTGGAAAGTACTTTACTTGTACCAACATTAATGGCATTT
CTTGCAAAAAGTGCATTAGTTGAAAAGTACGATTTATCGCACTTAAAAGAAATTGCATCTGGTGGC
GCACCTTTATCAAAAGAAATTGGGGAGATGGTGAAAAAACGGTTTAAATTAAACTTTGTCAGGCA
AGGGTATGGATTAACAGAAACCACTTCGGCTGTTTTAATTACACCGAAAGGTGTT[5]TAAACGAATT
CCTCGAGCGGGATCAATTCCGCCCCCCCCCTAACGTTACTGGCCGAAGCCGCTTGGAATAAGG
CCGGTGTGCGTTTGTCTATATGTTATTTTCCACCATATTGCCGTCTTTTGGCAATGTGAGGGCCC
GGAAACCTGGCCCTGTCTTCTTGACGAGCATTCCTAGGGGTCTTTCCCCTCTCGCCAAAGGAAT
GCAAGGTCTGTTGAATGTCGTGAAGGAAGCAGTTCCTCTGGAAGCTTCTTGAAGACAAACAACGT

---

[1] CMV enhancer and promoter
[2] Chimeric intron
[3] 30F-FFluc-358-544
[4] PLP2 cleavage site
[5] 30F-FFluc-4-354

CTGTAGCGACCCTTTGCAGGCAGCGGAACCCCCCACCTGGCGACAGGTGCCTCTGCGGCCAAA
AGCCACGTGTATAAGATACACCTGCAAAGGCGGCACAACCCCAGTGCCACGTTGTGAGTTGGAT
AGTTGTGGAAAGAGTCAAATGGCTCTCCTCAAGCGTATTCAACAAGGGGCTGAAGGATGCCCAG
AAGGTACCCCATTGTATGGGATCTGATCTGGGGCCTCGGTGCACATGCTTTACATGTGTTTAGTC
GAGGTTAAAAAACGTCTAGGCCCCCCGAACCACGGGGACGTGGTTTTCCTTTGAAAAACACGA
TAATA[6]CTTAATTAAGATGGCTTCCAAGGTGTACGACCCCGAGCAACGCAAACGCATGATCACTG
GGCCTCAGTGGTGGGCTCGCTGCAAGCAAATGAACGTGCTGGACTCCTTCATCAACTACTATGA
TTCCGAGAAGCACGCCGAGAACGCCGTGATTTTTCTGCATGGTAACGCAGCCTCCAGCTACCTG
TGGAGGCACGTCGTGCCTCACATCGAGCCCGTGGCTAGATGCATCATCCCTGATCTGATCGGAA
TGGGTAAGTCCGGCAAGAGCGGGAATGGCTCATATCGCCTCCTGGATCACTACAAGTACCTCAC
CGCTTGGTTCGAGCTGCTGAACCTTCCAAAGAAAATCATCTTTGTGGGCCACGACTGGGGGGCT
TGTCTGGCCTTTCACTACTCCTACGAGCACCAAGACAAGATCAAGGCCATCGTCCATGCTGAGA
GTGTCGTGGACGTGATCGAGTCCTGGGACGAGTGGCCTGACATCGAGGAGGATATCGCCCTGA
TCAAGAGCGAAGAGGGCGAGAAAATGGTGCTTGAGAATAACTTCTTCGTCGAGACCATGCTCCC
AAGCAAGATCATGCGGAAACTGGAGCCTGAGGAGTTCGCTGCCTACCTGGAGCCATTCAAGGAG
AAGGGCGAGGTTAGACGGCCTACCCTCTCCTGGCCTCGCGAGATCCCTCTCGTTAAGGGAGGC
AAGCCCGACGTCGTCCAGATTGTCCGCAACTACAACGCCTACCTTCGGGCCAGCGACGATCTGC
CTAAGATGTTCATCGAGTCCGACCCTGGGTTCTTTTCCAACGCTATTGTCGAGGGAGCTAAGAAG
TTCCCTAACACCGAGTTCGTGAAGGTGAAGGGCCTCCACTTCAGCCAGGAGGACGCTCCAGATG
AAATGGGTAAGTACATCAAGAGCTTCGTGGAGCGCGTGCTGAAGAACGAGCAGACCTAA[7]GCTA
GAGTCGACCTGCAGGCATGCAAGCTTGATATCAAGCTTATCGATAATCAACCTCTGGATTACAAA
ATTTGTGAAAGATTGACTGGTATTCTTAACTATGTTGCTCCTTTTACGCTATGTGGATACGCTGCT
TTAATGCCTTTGTATCATGCTATTGCTTCCCGTATGGCTTTCATTTTCTCCTCCTTGTATAAATCCT
GGTTGCTGTCTCTTTATGAGGAGTTGTGGCCCGTTGTCAGGCAACGTGGCGTGGTGTGCACTGT
GTTTGCTGACGCAACCCCCACTGGTTGGGGCATTGCCACCACCTGTCAGCTCCTTTCCGGGACT
TTCGCTTTCCCCCTCCCTATTGCCACGGCGGAACTCATCGCCGCCTGCCTTGCCCGCTGCTGGA
CAGGGGCTCGGCTGTTGGGCACTGACAATTCCGTGGTGTTGTCGGGGAAATCATCGTCCTTTCC
TTGGCTGCTCGCCTGTGTTGCCACCTGGATTCTGCGCGGGACGTCCTTCTGCTACGTCCCTTCG
GCCCTCAATCCAGCGGACCTTCCTTC[8]CCAATTCTACCGGGTAGGGGAGGCGCTTTTCCCAAGG
CAGTCTGGAGCATGCGCTTTAGCAGCCCCGCTGGGCACTTGGCGCTACACAAGTGGCCTCTGG
CCTCGCACACATTCCACATCCACCGGTAGGCGCCAACCGGCTCCGTTCTTTGGTGGCCCCTTCG
CGCCACCTTCTACTCCTCCCCTAGTCAGGAAGTTCCCCCCCGCCCCGCAGCTCGCGTCGTGCA
GGACGTGACAAATGGAAGTAGCACGTCTCACTAGTCTCGTGCAGATGGACAGCACCGCTGAGCA
ATGGAAGCGGGTAGGCCTTTGGGGCAGCGGCCAATAGCAGCTTTGCTCCTTCGCTTTCTGGGCT
CAGAGGCTGGGAAGGGGTGGGTCCGGGGGCGGGCTCAGGGGCGGGCTCAGGGGCGGGGCG
GGCGCCCGAAGGTCCTCCGGAGGCCCGGCATTCTGCACGCTTCAAAAGCGCACGTCTGCCGCG
CTGTTCTCCTCTTCCTCATCTCCGGGCCTTTCG[9]ACCTGCAGCCCAAGCTTACCATGACCGAGTA
CAAGCCCACGGTGCGCCTCGCCACCCGCGACGACGTCCCCAGGGCCGTACGCACCCTCGCCG
CCGCGTTCGCCGACTACCCCGCCACGCGCCACACCGTCGATCCGGACCGCCACATCGAGCGG
GTCACCGAGCTGCAAGAACTCTTCCTCACGCGCGTCGGGCTCGACATCGGCAAGGTGTGGGTC
GCGGACGACGGCGCCGCGGTGGCGGTCTGGACCACGCCGGAGAGCGTCGAAGCGGGGGCGG
TGTTCGCCGAGATCGGCCCGCGCATGGCCGAGTTGAGCGGTTCCCGGCTGGCCGCGCAGCAA
CAGATGGAAGGCCTCCTGGCGCCGCACCGGCCCAAGGAGCCCGCGTGGTTCCTGGCCACCGT
CGGCGTCTCGCCCGACCACCAGGGCAAGGGTCTGGGCAGCGCCGTCGTGCTCCCCGGAGTGG
AGGCGGCCGAGCGCGCCGGGGTGCCCGCCTTCCTGGAGACCTCCGCGCCCCGCAACCTCCCC

[6] ECMV IRES
[7] Renilla luciferase
[8] WPRE
[9] PGK promoter

TTCTACGAGCGGCTCGGCTTCACCGTCACCGCCGACGTCGAGGTGCCCGAAGGACCGCGCACCTGGTGCATGACCCGCAAGCCCGGTGCCTGA[10]

[10] Puromycin resistance
