# Supplementary material for: A protease-activatable luminescent biosensor and reporter cell line for authentic SARS-CoV-2 infection
Source: PLoS Pathog. 2022 Feb 10;18(2):e1010265. doi: 10.1371/journal.ppat.1010265 (PMC8865646; doi:10.1371/journal.ppat.1010265)
Supplement: S2 Appendix — (PDF) [file ppat.1010265.s012.pdf]

S2 Appendix. Gene block for cloning of SARS-CoV Papain-like protease catalytic domain.

GCTTGGTACCGAGCTCG[1]GCCGCCACC[2]ATG[3]GAGGTGAAGACGATTAAGGTATTTACAACTGTC
GACAACACTAATCTTCACACACAGCTTGTTGATATGTCCATGACGTATGGTCAACAATTCGGACC
TACATACCTGGATGGAGCAGATGTCACCAAAATAAAGCCCCATGTAAATCATGAGGGCAAAACCT
TCTTTGTTTTGCCTTCTGATGATACCCTTAGGAGTGAAGCGTTCGAATATTATCATACTTTGGATG
AAAGTTTTTTGGGCCGCTACATGTCTGCCTTGAACCACACAAAAAAGTGGAAATTCCCTCAGGTA
GGAGGACTCACGTCAATCAAATGGGCCGACAATAATTGCTATTTGTCATCTGTGCTTCTTGCACT
GCAGCAGTTGGAAGTGAAATTTAATGCACCCGCTTTGCAGGAAGCCTACTACCGAGCTAGAGCC
GGGGATGCGGCCAATTTCTGCGCTCTTATTCTGGCTTACAGCAACAAGACTGTGGGCGAACTCG
GGGACGTGCGGGAGACCATGACACATCTTTTGCAGCACGCGAACTTGGAAAGCGCCAAGCGGG
TTTTGAACGTCGTGTGCAAACATTGCGGGCAGAAGACGACGACTCTTACGGGGGTCGAAGCCGT
GATGTATATGGGAACATTGTCATATGACAATCTCAAAACCGGGGTTAGCATCCCGTGTGTTTGTG
GCAGGGATGCTACGCAATATCTTGTTCAACAAGAGTCTAGCTTTGTCATGATGTCCGCCCCTCCA
GCTGAGTACAAACTTCAGCAAGGAACTTTTCTCTGCGCTAACGAGTACACAGGTAATTATCAGTG
TGGTCACTACACTCATATAACAGCAAAGGAAACCTTGTACCGAATCGATGGTGCGCACCTGACAA
AAATGAGCGAATATAAGGGTCCAGTTACAGATGTCTTCTACAAAGAGACATCCTATACCACGACT
ATAAAG[4]TAA[5]CTGCAGATATCCATCACAC[6]

---

[1] Overlap with BamHI/EcoRI
[2] Kozak sequence
[3] Start codon
[4] Codon-optimized SARS-CoV PLPro catalytic domain
[5] Stop codon
[6] Overlap with BamHI/EcoRI
