# Supplementary material for: A protease-activatable luminescent biosensor and reporter cell line for authentic SARS-CoV-2 infection
Source: PLoS Pathog. 2022 Feb 10;18(2):e1010265. doi: 10.1371/journal.ppat.1010265 (PMC8865646; doi:10.1371/journal.ppat.1010265)
Supplement: Attachment — Submitted filename: Response to reviewers_18 11 2021_v5.pdf [file ppat.1010265.s014.pdf]

UNIVERSITY OF CAMBRIDGE
DEPARTMENT OF MEDICINE
CAMBRIDGE INSTITUTE OF THERAPEUTIC IMMUNOLOGY AND INFECTIOUS DISEASE
CAMBRIDGE BIOMEDICAL CAMPUS, HILLS RD, CAMBRIDGE, CB2 0XY
Nicholas J. Matheson PhD, FRCP
MRC Clinician Scientist Fellow and University Lecturer in Virology
Honorary Consultant in Infectious Diseases
njm25@cam.ac.uk

18th Nov 2021

Dear Editors,

Many thanks for your supportive and constructive feedback, and for encouraging us to submit a revised version of our manuscript:

A protease-activatable luminescent biosensor and reporter cell line for authentic SARS-CoV-2 infection

We have given careful consideration to all the comments from you and the reviewers and have, we believe, addressed them all in full.

In particular, we have incorporated a number of additional experiments into several new or substantially revised figures and supporting figures, to confirm and extend our previous observations. These comprise:

- Extensively revised **Fig 5** and new supporting figure **S6 Fig**, showing the correlation between assays using our luminescent reporter cell line and a traditional plaque assay (relative quantitation of infectious virus and calculation of NT50s)
- Revised figure **Fig 4E** and new figure **Fig 5D**, showing data for the delta variant of SARS-CoV-2 (detection of viral replication and measurement of neutralizing antibody activity)
- New supporting figure **S5 Fig**, showing the kinetics of reporter cell activation
- New supporting figure **S7 Fig**, showing the reproducibility of assays using our luminescent reporter cell line

Please note that, some of the panels and supporting figures and appendices have been re-numbered as a consequence. In addition:

- We provide a supporting file **S13 File** including all numerical values used to generate graphs for the figures
- We have added an Author summary
- We have processed our figures using PACE, and include them as individual .tif files
- We have corrected a small number of typographical errors
- We are preparing a detailed laboratory protocol to deposit at protocols.io (or equivalent)

Please find below our detailed, point-by-point responses in plain text, with reviewers' comments *shaded/in italics*. Key textual changes to the manuscript are highlighted in yellow. Where indicated, line numbers refer to the revised manuscript.

Finally, we apologise again for the delay in submitting these revisions, which has arisen because of my ongoing commitments to the COVID-19 pandemic response at Cambridge University Hospitals and the University of Cambridge.

We hope that they satisfy you and the reviewers, and that our manuscript is now appropriate for publication in *PLoS Pathogens*.

Yours sincerely,

Nick Matheson (for the authors)

Point-by-point responses

General comments

Both reviewers appreciated the advance presented by this reporter cell line, but also raised concerns about the interpretation of the data as well as a need to confirm the use of this reporter cell line with a live virus assay. Some data which show how the metric reported by this reporter cell line corresponds in some way to more familiar metrics used in traditional PRNT or FFU reduction assays would broaden the impact and utility of this system for the community.

Thank you for the summary. As detailed below, we have now addressed all of the points relating to the interpretation of our data, and validated the use of our reporter cell line by comparison with a traditional Plaque Reduction Neutralisation Test (PRNT).

Part I - Summary

No responses were requested or required in respect of these kind words and supportive comments.

Part II – Major Issues: Key Experiments Required for Acceptance

Reviewer #1.

In the development of the initial strategy, what percentage of cells are transfected in fig 1 (BFP+)? The data and gating strategy should be shown as supplemental data.

Thank you for the suggestion. We had already provided an indicative gating strategy in **S2 Fig** (now **S2A Fig**). In addition, we now show the % BFP+ (transfected) cells for all experiments in **Fig 1** as **S2B Fig**.

In later studies the luciferase system is moved to a lentivirus approach and stable cells are generated. How stable is the luciferase activity over time and passage number?

Thank you for the question. Results from our reporter cell line are highly reproducible, and robust to both time in culture and passage number. We have added a new supporting figure **S7 Fig** to demonstrate this, and adjusted the text accordingly:

> Results, lines 194-6
>
> Activation of reporter cells was stable over multiple passages (**S7A Fig**), and NT50s for control serum determined using these cells were highly reproducible across independent experiments and over time (**S7B Fig**).

Additionally, in panel E of figure 4, the variant of concern B.1.1.7 is detected using the cells. In order to support the statement that the luminescent biosensor could be activated by different isolates, it may be advantageous to provide similar data for additional variates.

Thank you for the suggestion. We have revised **Fig 4E** to include new data showing reporter activation by a lineage B.1.617.2 (delta) variant of concern, and adjusted the text accordingly:

Results, lines 184-7

Nonetheless, assays may be conducted in a 96-well or 384-well plate format (**Fig 4D**), and – as expected based on PLP2 sequence conservation (**S1A Fig**) – cells are readily activated by different isolates of wildtype SARS-CoV-2, including the B.1.1.7 (alpha) and B.1.617.2 (delta) variants of concern (**Fig 4E**).

In addition, we have added a new figure **Fig 5D**, comparing neutralising activity of serum samples from vaccinated controls against lineage B, B.1.1.7 (alpha) and B.1.617.2 (delta) variants, and adjusted the text accordingly:

Results, lines 203-5

Finally, and again in agreement with previous observations [18], we observed a modest yet statistically significant reduction in neutralising activity against the B.1.617.2 (delta) variant of concern (**Fig 5D**).

Finally, please note that the Papain-Like Protease cleavage site exploited by the luminescent biosensor (PLP2) is highly conserved across SARS-CoV as well as SARS-CoV-2 (**S1A Fig**) viruses, and the biosensor may therefore be activated by the equivalent SARS-CoV protease (**S1B Fig**).

In the final figure of the paper (fig 5), neutralizing activity as measured with the reporter cell line for human serum post vaccination is shown. While these data illustrate the efficacy of the system, it would be necessary to compare the neutralization curves as measured with the reporter cell line to those generated using widely-utilized assays, such as PRNT or FRNT.

Thank you for the suggestion. We have extensively revised **Fig 5** and added a new supporting figure **S6 Fig**, showing the correlation between assays using our reporter cell line and a traditional Plaque Reduction Neutralisation Test (PRNT). We agree that adding this data significantly strengthens the paper, and have adjusted the text accordingly:

Results, lines 188-94

To demonstrate the utility of our luminescent reporter cell line for measuring SARS-CoV-2 neutralising activity, we tested serum samples from 5 healthy control donors 21 days after their first or second doses of Pfizer-BioNTech BNT162b2 mRNA vaccine using both clone B7 reporter cells and Plaque Reduction Neutralisation Tests (PNRTs) in VeroE6 cells (**Fig 5A-C** and **S6 Fig**). Similar neutralisation curves were obtained from both assays (**Fig 5A**), with a striking correlation between the calculated neutralising titres at 50% inhibition (NT50s) (**Fig 5B**).

Reviewer #2.

Quantitation of infectious virus - while the authors clearly demonstrate the use of their biosensor for measuring neutralising antibodies or the effects of drugs on viral replication, it is less clear how their system can be used for quantification of infectious virus. Given the lack of absolute quantification using luminescent readouts as well as the timing at which the assay is performed which will include multiple rounds of infection, no absolute quantification of viral stock can be achieved with this system as opposed to plaque assays. For example, when comparing variants it would not be possible to distinguish whether a different readout at 24 hours is due to different amounts of infectious units at time of infection or due to different replication dynamics.
This should be accurately stated in the manuscript and be rephrased in line 32 in the abstract ("quantitation of infectious virus") and line 145-146 ("quantitation of infectious virus").

Thank you for the comments and suggestions, with which we agree. We have therefore rephrased the specific cases highlighted by the reviewer (Abstract, line 33 and Results, line 152 in the revised manuscript) to state "relative quantitation", and reviewed and/or revised other similar examples elsewhere in the manuscript to confirm that the usage is always appropriate. In addition, we have added a statement about this to the text:

Results, lines 180-4

Because the luminescent signal at 24 h is a continuous readout dependent on both the starting inoculum and the rate of spreading infection (leading to an increase in the number of infected cells), the FFluc/Rluc ratio cannot be used directly for the absolute quantitation of infectious units in viral stocks (unlike a plaque assay).

Quantitation of replication - the authors state at several points in the manuscript that their reporter system quantitates "viral replication" (line 29, line 49, line 143-14, line 172, line 203), when "viral infection" would be more accurate. The experiment presented in Fig. 3F-G are indeed indicative of viral replication; however given the timing of events and the mechanisms of action for remdesivir and GC 376 which do not block infection but replication inside the cell, one would expect that initially the luciferase sensor should still get activated as PLPro expression precedes the viral mechanisms that are inhibited by either drug.
It could therefore be useful to provide some temporal dynamics for the luminescent sensor, e.g. how quickly after infection can an increase in luminescence be detected and for how long can that initial signal be detected (e.g. by adding neutralising antibodies after infection to prevent secondary infection).

Thank you for the comments and suggestions, with which we agree. We have therefore reviewed and/or revised all statements about "viral replication" in the manuscript to confirm that the usage is appropriate. In addition, we have added a new supporting figure **S5A-D Fig**, showing the temporal dynamics of luminescent biosensor activation, and adjusted the text accordingly:

Results, lines 178-80

Using clone B7 reporter cells, an increase in luminescence is readily detectable by 12 h post-infection, and the FFluc/Rluc ratio correlates closely with the frequency of spike-positive (infected) cells over a 24 h time course (**S5A-D Fig**).

We agree that specifically preventing secondary (spreading) infection subsequent to seeding of the first round could, in theory, provide useful information. As reported by others, however – SARS-CoV-2 cell-to-cell spread occurs rapidly and is insensitive to antibody neutralization. Jackson, Sigal et al. bioRxiv 2021.06.01.446516; https://doi.org/10.1101/2021.06.01.446516 – we find that spreading infection is incompletely attenuated by the delayed addition of neutralising antibodies. The final experiment suggested is therefore not currently technically possible.

Availability of materials - the authors should evaluate whether they can make the generated reporter clone more easily available to the community for example by depositing with BEI resources (https://www.beiresources.org/Home.aspx) or a similar repository.

Thank you for the suggestion. Our aim is to make our clone B7 reporter cells as easily available to the community as possible. The UK National Institute for Biological Standards and Control (NIBSC) repository (https://www.nibsc.org/) has therefore kindly agreed to distribute the cells:

From: CFAR <cfar@nibsc.org>
Sent: 05 October 2021 18:25
To: Nicholas Matheson <njm25@cam.ac.uk>
Subject: RE: Request to deposit COVID-19 research material - reporter cell line

Dear Nicholas,

Thank you for contacting us. I would be very grateful if you were willing to deposit your reporter cell line to our repository. I am not aware of any other luciferase reporter cell line for SARS-CoV-2 and I believe this tool will be very useful for the scientific community. This will be a great addition to our current SARS-CoV-2 susceptible cell lines.

I have attached our deposit MTA (NIBSC - depositor) and our standard outgoing MTA (NIBSC - end-user), please share both documents with your TTO and ask them to complete and sign the deposit MTA. Any questions, please ask. Many thanks again for depositing your reagent to NIBSC.

Best regards,

Yann

Yann Le Duff, PhD
Centre for AIDS Reagents (CFAR)
National Institute for Biological Standards and Control (NIBSC)
Blanche Lane, South Mimms, Potters Bar, Hertfordshire, EN6 3QG, UK
Telephone: +44 (0) 1707641221

We are currently arranging the necessary MTAs and physical transfer of materials, and have adjusted our statement about the availability of materials accordingly:

Materials and methods, lines 557-60

Biological materials from this study are available from specified commercial sources, or from the corresponding author on execution of an appropriate Material Transfer Agreement (MTA). Clone B7 reporter cells will also be made available via the National Institute for Biological Standards and Control (NIBSC) repository (https://www.nibsc.org/).

Part III – Minor Issues: Editorial and Data Presentation Modifications

Reviewer #1.

An introduction to SARS-CoV-2 proteases should be included to provide greater context.

Thank you for the suggestion. We have consolidated and extended our introduction to SARS-CoV-2 proteases to form the 3rd paragraph of the Introduction:

Introduction, lines 73-83

During the SARS-CoV-2 replication cycle, the 30 kb single-stranded positive-sense genomic RNA is used as a template to generate the polyproteins 1a and 1ab (pp1a and pp1ab). In turn, these polyproteins are processed into 16 non-structural proteins (nsp1 to nsp16) by the action of two virally-encoded proteases on sequence-specific cleavage sites: Papain-like Protease (PLPro, or nsp3), which cleaves nsp1, nsp2, and nsp3, and Main or 3C-like Protease (MPro, or nsp5), which cleaves the remaining non-structural proteins [8]. Both proteases contribute to the assembly of the viral replication and transcription complex (RTC), making them attractive targets for drug development. At the same

time, we show here that the expression of SARS-CoV-2 protease activity during viral replication may be exploited for the detection and quantitation of infected cells, and demonstrate the utility of this approach for assays of candidate antivirals and neutralising antibodies.

Reviewer #2.

What was the rationale for choosing clone B7 over G7, which had a stronger fold change upon infection?

Thank you for the question. Clone B7 was selected because it was morphologically identical to parental HEK293T cells, whereas clone G7 tended to grown in clumps. We have adjusted the text accordingly:

Results, lines 175-7

Clone B7 was selected for the remaining experiments shown in this paper, because it was morphologically identical to parental HEK293T cells, whereas clone G7 tended to grow in clumps.

For the purpose of comparison, it should be stated what MOI the viral dilutions correspond to in the experiments presented in Figure 3C-D and Figure 4C-D.

Thank you for the suggestion. We have added MOI equivalents to the figure captions for **Fig 3C-D** and **Fig 4C-E**.

Buchrieser et al. 2020 EMBO J should be cited as demonstrating syncytia formation and use of this phenomenon to detect viral infection using their "S-Fuse" cells.

Thank you for the suggestion. We have adjusted the text accordingly:

Results, lines 115-7

Whilst syncytia are lost during flow cytometric analysis (S3B-C Fig), their formation has previously been exploited for the quantitation of SARS-CoV-2 infection by high-content imaging [11].
